# The effects of hyperuricemia on endothelial cells are mediated via GLUT9 and the JAK2/STAT3 pathway

**DOI:** 10.1007/s11033-021-06840-w

**Published:** 2021-10-30

**Authors:** Qian Nie, Miaomiao Liu, Zhimei Zhang, Xuemei Zhang, Chao Wang, Guangyao Song

**Affiliations:** 1 Department of Internal Medicine, Hebei Medical University, 361 Zhongshan East Road, Shijiazhuang, 050017 China; 2 Physical Examination Center of Hebei General Hospital, 348 Heping West Road, Shijiazhuang, China; 3 Hebei Key Laboratory of Metabolic Diseases, Hebei General Hospital, 348 Heping West Road, Shijiazhuang, China; 4 Department of Oncology, Hebei General Hospital, 348 Heping West Road, Shijiazhuang, China; 5 Department of Rheumatism and Immunology, Hebei General Hospital, 348 Heping West Road, Shijiazhuang, China

**Keywords:** Uric acid, Endothelial cell, GLUT9, Endothelial nitric oxide synthase, JAK2/STAT3 pathway

## Abstract

**Background:**

Uric acid (UA) transporters mediate the uptake and outflow of UA, and are greatly involved in the control of UA concentrations. Glucose transporter 9 (GLUT9), one of the UA transporters, has been confirmed to be expressed in human umbilical vein endothelial cells (HUVECs). This study aimed to characterize GLUT9’s effect on intracellular UA accumulation in HUVECs in a high-UA environment and to explore the mechanism of cellular dysfunction.

**Methods and results:**

HUVECs were treated with UA to establish a model of cellular dysfunction. Then, UA uptake, GLUT9 expression and endothelial nitric oxide synthase (eNOS) and reactive oxygen species (ROS) amounts were measured. UA uptake was concentration- and time-dependent, and UA treatment significantly reduced nitric oxide (NO) levels and eNOS activity. UA also upregulated pro-inflammatory molecules and GLUT9, and increased intracellular ROS amounts in HUVECs. GLUT9 knockdown reduced UA uptake and ROS content, but antioxidant treatment did not reduce GLUT9 expression. To assess the function of JAK2/STAT3 signaling, HUVECs were treated with UA, and the phosphorylation levels of JAK2, STAT3, IL-6 and SOCS3 were increased by a high concentration of UA. In addition, GLUT9 knockdown reduced the phosphorylation of JAK2/STAT3 intermediates and increased p-eNOS amounts.

**Conclusions:**

GLUT9 mediated the effects of high UA levels on HUVECs by increasing the cellular uptake of UA, activating JAK2/STAT3 signaling, and reduced the production of active eNOS and NO in HUVECs.

**Supplementary Information:**

The online version contains supplementary material available at 10.1007/s11033-021-06840-w.

## Introduction

Uric acid (UA) constitutes the end-product of primates’ metabolic reactions transforming purine nucleosides. Although at normal concentrations, UA plays antioxidative roles in humans, epidemiological evidence indicates abnormally high UA concentrations are associated with cardiovascular abnormalities, such as hypertension, coronary heart disease, and atherosclerosis [1–4]. However, the underpinning pathophysiological mechanisms require further elucidation. Endothelial dysfunction is the initial defect leading to cardiovascular disease [5]. Recently, animal experiments have demonstrated UA reduces endothelial nitric oxide synthase (eNOS) activity as well as nitric oxide (NO) amounts by inducing mitochondrial calcium overload, endoplasmic reticulum (ER) stress and xanthine oxidase activation in human umbilical vein endothelial cells (HUVECs), which results in apoptosis and endothelial dysfunction [6–8].

Serum UA concentration mainly depends on the dynamic balance between UA production and its excretion. UA transporters, including URAT1, GLUT9 and breast cancer resistance protein (BCRP), mediate UA uptake and outflow, greatly regulating UA concentrations in the body [9, 10]. It was previously demonstrated UA uptake level also affects the degree of inflammation [3, 11]. Reactive oxygen species (ROS) concentrations are increased by UA, which can reduce the expression of BCRP in HUVECs and decrease UA efflux, leading to endothelial dysfunction [12]. GLUT9 expression in HUVECs has also been confirmed by previous studies and our preliminary experiments. Therefore, we hypothesized that the balance between intracellular and extracellular UA amounts is regulated by uric acid transport, which should also involve the role of GLUT9. In hyperuricemia, UA transporters increase its intracellular accumulation, thereby promoting cell dysfunction. To test this hypothesis, the effects of high UA concentration on UA’s intracellular accumulation and GLUT9 levels in HUVECs were examined, exploring the mechanism by which UA induces cell damage.

## Methods

### Cell culture

HUVECs provided by the China Center for Type Culture Collection (CCTCC, China) underwent culture in F12-K basal medium supplemented with 10% FBS, ECGS and 0.1 mg/ml heparin sodium at 37 °C under 5% CO_2_. UA (Sigma, USA) was dissolved in serum-free F12-K medium, incubated at 37 °C with constant shaking, and filtered using 0.22-μm syringe filters (Millipore, USA) [11]. The UA concentration did not decrease when the urate-medium was stored at 4 °C within 72 h. Therefore, only the urate-medium prepared within 72 h was used. A polarizing microscope (Olympus, Japan) detected no UA crystals during cell treatments.

### Assessment of the cytotoxicity of uric acid

HUVECs were cultured in 96-well plates and administered diverse amounts of UA (0, 5, 10, 15 and 20 mg/dl) at ~ 80% confluence. Cell Counting Kit-8 (Dojindo, Japan) was utilized to assess cell viability as directed by the manufacturer.

### Measurement of uric acid uptake

UA uptake assays were performed in serum-free F12-K medium containing various amounts of UA (5, 10, 15 and 20 mg/dl). A Uric Acid Assay Kit (Sigma) was then utilized for the measurement of UA amounts in the cell culture supernatant after incubation for 24 h and 48 h, as directed by the manufacturer. Next, cell lysis was performed after cell counting with a hemocytometer. UA uptake was quantified by normalizing UA concentration to the cell count.

### NO release assessment

NO release was measured in the cell culture supernatant using a Total Nitric Oxide Assay Kit (Beyotime Biotech, China), as directed by the manufacturer. This was achieved by measuring nitrite and nitrate amounts with the Griess reagent. Absorbance was measured on a microplate reader at 520 nm after color development.

### Measurement of eNOS

The concentration of eNOS in the cell culture supernatant was assessed with a Human eNOS ELISA kit (Beyotime Biotech), as directed by the manufacturer. Briefly, eNOS in the supernatant was bound by an anti-eNOS antibody in an ELISA plate, and then horseradish peroxidase-labeled avidin and the developing agent were added in sequence. Absorbance at 520 nm was used to calculate eNOS concentration.

### Measurement of intracellular ROS concentration

Intracellular ROS concentration was assessed with DCFH-DA (Sigma). The cells underwent seeding in 35-mm plates at 4 × 10^4^ cells/ml, stimulated with UA. The culture medium was replaced with DCFH-DA diluted in serum-free medium to 10 µM, followed by a 20-min incubation. After three washes with serum-free medium, fluorescence was immediately assessed on a Leica fluorescence microscope (Leica, Germany), with excitation and emission at 488 nm and 525 nm, respectively.

### Quantitative real-time PCR

RNA extraction utilized Transzol reagent (TransGen Biotech, China), and RNA purity was determined by absorbance measurements at 260 and 280 nm. Reverse transcription was carried out with a Reverse Transcriptase kit (TransGen Biotech), and transcript abundance was quantitated on a 7500 Real-Time PCR System (Applied Biosystems, USA) using the SYBR Green detection system (TransGen Biotech).

Target gene expression was normalized to *Gapdh* mRNA amounts. The 2^−ΔΔCt^ method was utilized for data analysis. The primers used are listed in Table S1.

### Western blot

Lysates were prepared from HUVECs in the RIPA buffer supplemented with protease and phosphatase inhibitors (Solarbio, China). The BCA kit (Thermo Fisher Scientific) was utilized for protein quantitation. Equal amounts of total protein underwent separation by sodium dodecyl sulphate (SDS)-polyacrylamide gel electrophoresis (PAGE), followed by electro-transfer unto PVDF membranes (Millipore, USA). Upon blocking with 5% skim milk (2 h at ambient), successive incubations were carried out with primary (overnight at 4 °C) and secondary (50 min at ambient) antibodies. After washing, detection of immunoreactive bands utilized the enhanced chemiluminescence reagent (Thermo Fisher Scientific). ImageJ was used for imaging and quantitation. Target protein expression was normalized to that of GAPDH. The utilized antibodies, provided by Abcam (UK), Proteintech (USA) or Cell Signaling (USA), were directed against GLUT9 (1:1000), eNOS (1:1000), phosphorylated (p)-eNOS (1:1000), total (t)-JAK2 (1:1500), p-JAK2 (1:1000), t-STAT3 (1:1500), p-STAT3 (1:1000), IL-6 (1:2000), SOCS3 (1:2000) and GAPDH (1:20,000).

### Immunofluorescence (IF)

HUVECs underwent fixation with 4% formalin in PBS and permeabilization with 0.3% Triton X-100 for 10 min. Upon blocking with 3% BSA for 30 min, incubation was carried out with mouse anti-p-eNOS (1:500) and rabbit anti-GLUT9 (1:500) antibodies, respectively, overnight at 4 °C in a humidified environment. After washing, the cells were covered with secondary antibodies and incubated at ambient for 50 min. DAPI counterstaining was performed, and cells were finally examined under a Leica fluorescence microscope (Leica).

### Effects of GLUT9 siRNA transfection into HUVECs

Small interfering (si)RNAs targeting SLC2A9 (GLUT9), shown in Table S2, were designed and produced by GenePharma (China). A 20-μM siRNA stock solution was prepared as directed by the manufacturer. Then, the transfection complex was prepared by mixing Opti-MEM (Thermo Fisher Scientific, 200 µl), siRNA stock solution (5 µl) and siRNA-Mate transfection reagent (GenePharma, 10 µl). HUVECs were transfected using these transfection complexes with siRNA concentration of 50 nM in a 6-well plate at 37 °C for 24 h. Next, UA was supplemented to appropriate wells, and cells were subsequently collected to measure transfection efficiency by RT-PCR and immunoblot. *N*-acetylcysteine (NAC, 10 mM; Beyotime), an antioxidant, was supplemented to some wells for determining the impact of oxidative stress on GLUT9 expression.

### Statistical analysis

SPSS 22.0 (SPSS, USA) was utilized for data analysis. Data are mean ± SD. Group pairs and multiple groups were compared by Student’s *t*-test and one-way ANOVA for normally distributed data. Otherwise, a non-parametric test was applied. *P* < 0.05 was deemed to reflect statistical significance.

## Results

### Effects of UA on survival and UA uptake in HUVECs

**Fig. 1:**
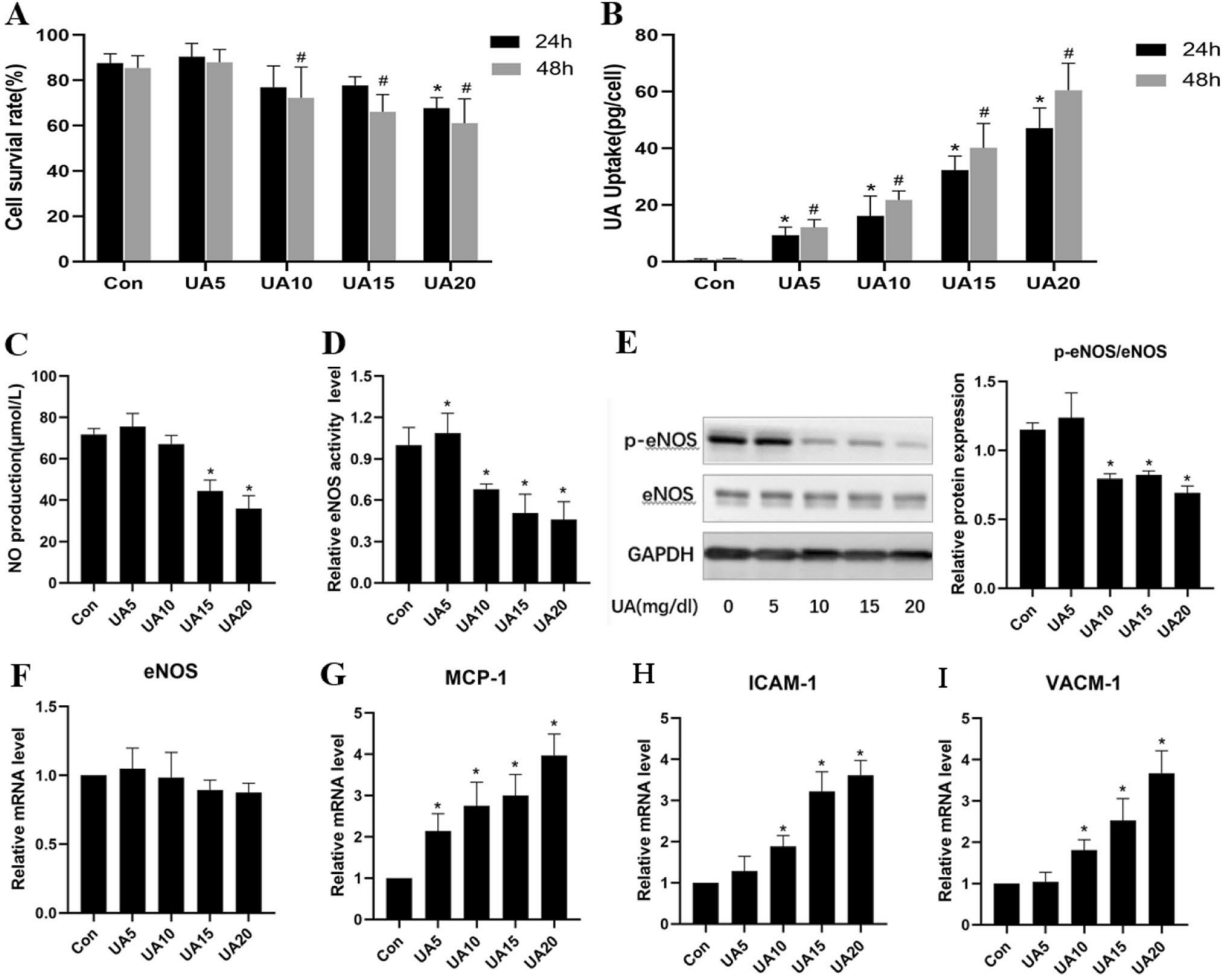
UA’s effects on uric acid uptake, NO and eNOS amounts and inflammation in HUVECs. **A** Cell survival was measured using CCK-8 following incubation with 5, 10, 15, or 20 mg/dl UA. **B** Effect of treatment with the indicated concentrations of urate on UA uptake by HUVECs. *P < 0.05 UA groups versus Con (24 h); ^#^*P* < 0.05 UA groups versus Con (48 h). **C** NO production, measured with Griess reagent. **D**–**F** eNOS concentration in the cell culture supernatant, and relative protein and mRNA expression, measured using an eNOS assay kit, real-time PCR and western blot analysis, respectively. **G**–**I** MCP-1, ICAM-1 and VCAM-1 mRNA amounts. *P < 0.05 UA group versus Con. *Con* control group, *UA5* uric acid 5 mg/dl, *UA10* uric acid 10 mg/dl, *UA15* uric acid 15 mg/dl, *UA20* uric acid 20 mg/dl, *HUVEC* human umbilical vein endothelial cell, *NO* nitric oxide, *eNOS* endothelial nitric oxide synthase. Data are mean ± SD (n = 3)

UA at 5, 10 and 15 mg/dl had no clear effect on cell survival, but decreased survival was detected at a UA concentration of 20 mg/dl (Fig. 1A). The uptake of UA increased concentration- and time-dependently; thus, high UA concentrations were associated with elevated uptake by endothelial cells (Fig. 1B).

### Uric acid causes concentration-dependent dysfunction and inflammation in HUVECs

As shown in Fig. 1C, D, NO concentration and eNOS activity in the cell culture supernatant were significantly reduced by 15 mg/dl and 20 mg/dl UA. The gene and protein expression levels of eNOS were similar in all treatment groups, but eNOS phosphorylation was significantly lower in UA-treated cells (Fig. 1E, F), indicating impaired endothelial cell function. In addition, qRT-PCR analysis of proinflammatory factors (MCP-1, ICAM-1 and VCAM-1) showed that UA also increased their expression dose-dependently in HUVECs (Fig. 1G–I).

### The effect of GLUT9 on UA uptake and oxidative stress in HUVECs

**Fig. 2:**
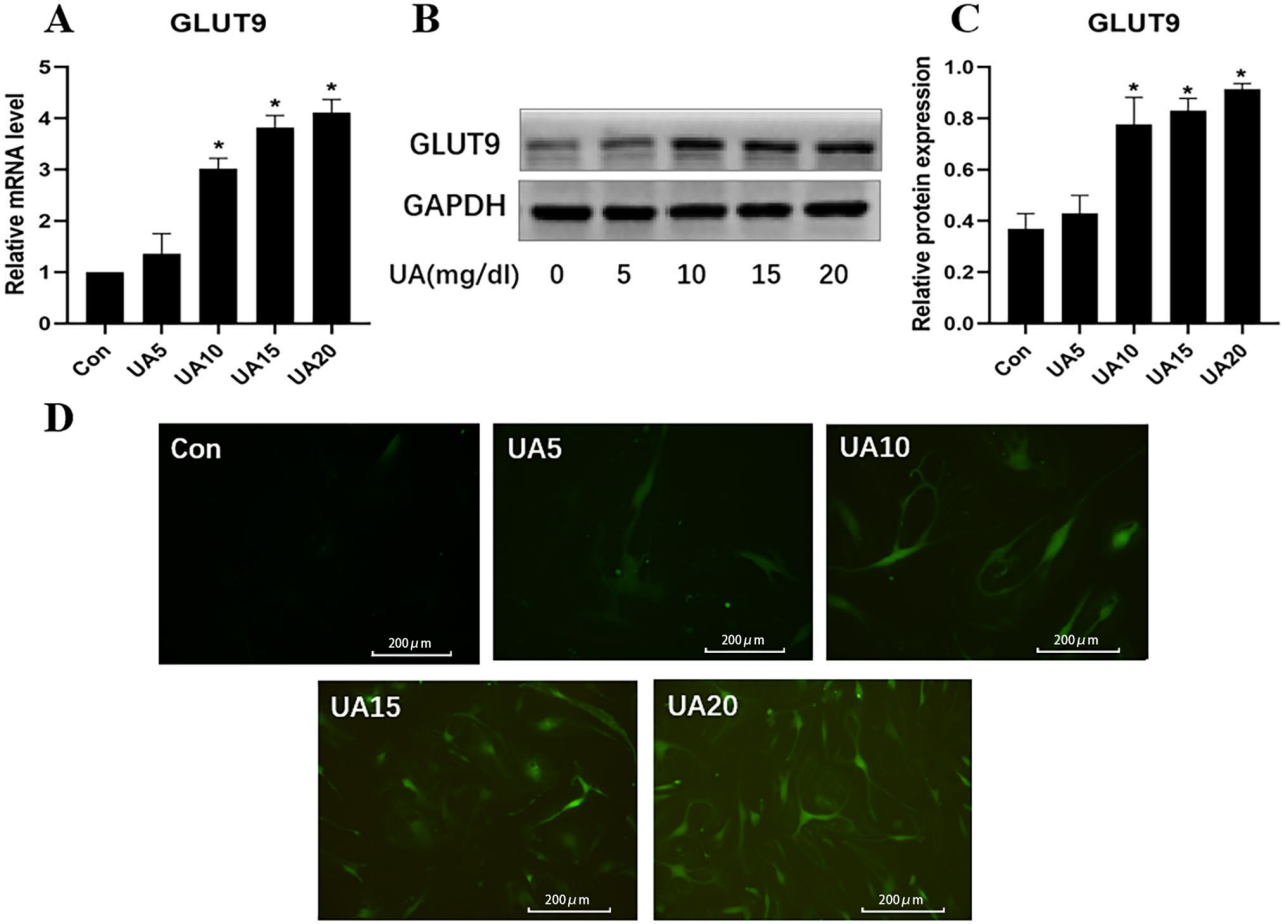
The role of GLUT9 in urate uptake by and oxidative stress in HUVECs. **A**–**C** Relative GLUT9 mRNA and protein expression in cells administered different UA amounts. **D** Impact of UA at various concentrations on intracellular reactive oxygen species (ROS) content, assessed using the specific probe DCFH-DA (scale bars 200 μm). *P < 0.05 UA group versus Con. Data are mean ± SE (n = 3)

The expression of GLUT9 was measured by qRT-PCR and immunoblot. The results revealed GLUT9 mRNA and protein amounts were markedly increased after administration of 10, 15 and 20 mg/dl UA, respectively, compared with control cells (Fig. 2A–C). In addition, these concentrations of UA increased intracellular ROS content (Fig. 2D).

**Fig. 3:**
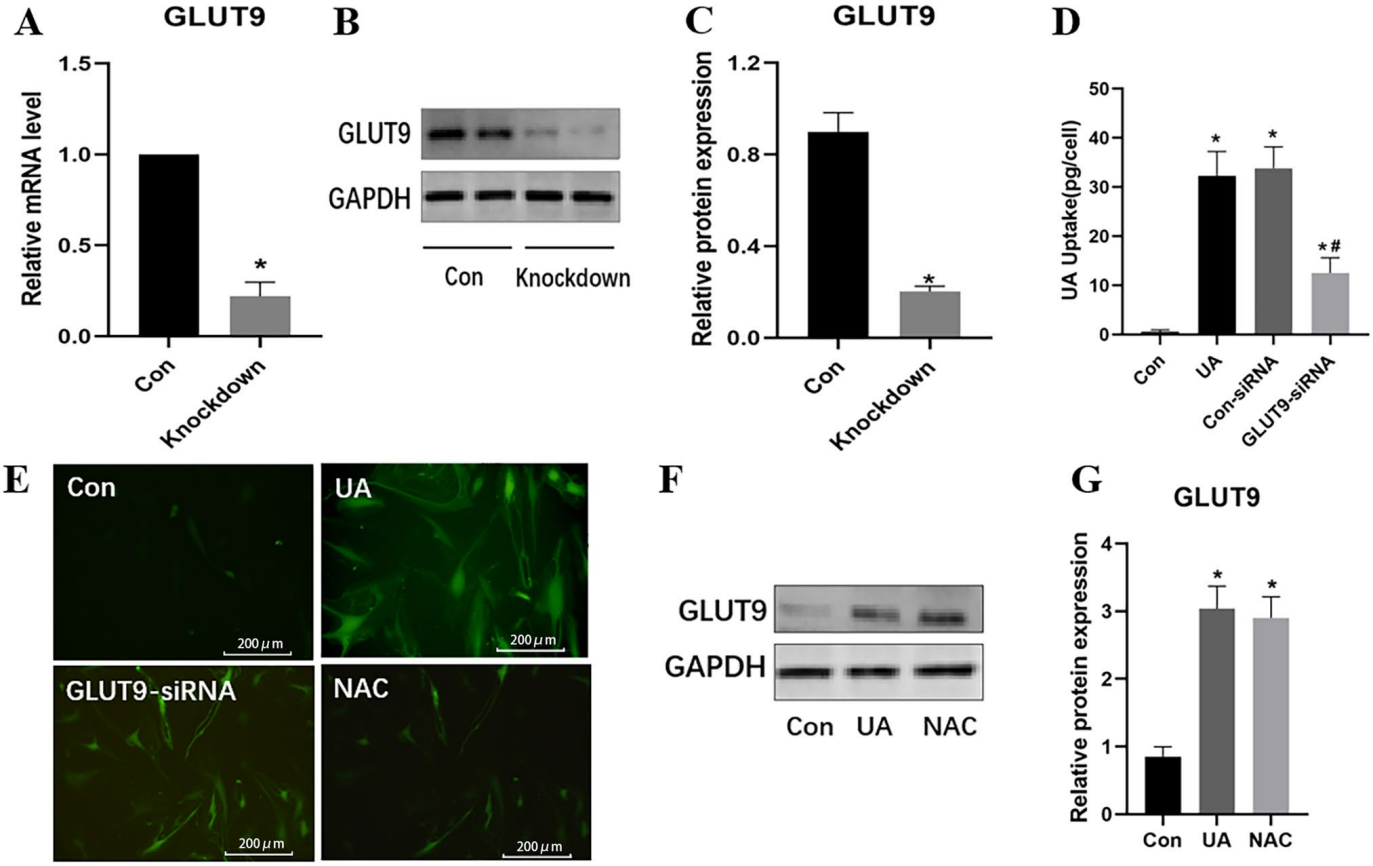
Effects of GLUT9 knockdown on urate uptake by and oxidative stress in HUVECs. **A**–**C** The mRNA and protein amounts of GLUT9 after GLUT9 siRNA transfection. **D** Effect of GLUT9 knockdown on urate uptake by HUVECs. **E** Effect of GLUT9 knockdown and *N*-acetylcysteine (NAC) treatment on intracellular ROS content (scale bars 200 μm). **F** and **G** Effect of NAC on GLUT9 protein expression in HUVECs. *P < 0.05 versus Con. ^#^*P* < 0.05 versus UA. Data are mean ± SE (n = 3)

To confirm that GLUT9 affects UA uptake by HUVECs, GLUT9 siRNA was transfected into these cells. This caused marked reductions in GLUT9 mRNA and protein amounts, by 78.90% and 77.47%, respectively, indicating a high transfection efficiency (Fig. 3A–C). Knockdown of GLUT9 also significantly reduced UA uptake (*P* < 0.05) (Fig. 3D). Next, to determine the role of GLUT9 in oxidative stress, cells were treated with siRNA-GLUT9 and/or the antioxidant NAC. siRNA-GLUT9 transfection reduced ROS content in cells (Fig. 3E). While NAC inhibited ROS production, it did not inhibit GLUT9 expression (Fig. 3F, G).

### Uric acid causes oxidative stress in HUVECs by inducing JAK2/STAT3 signaling

**Fig. 4:**
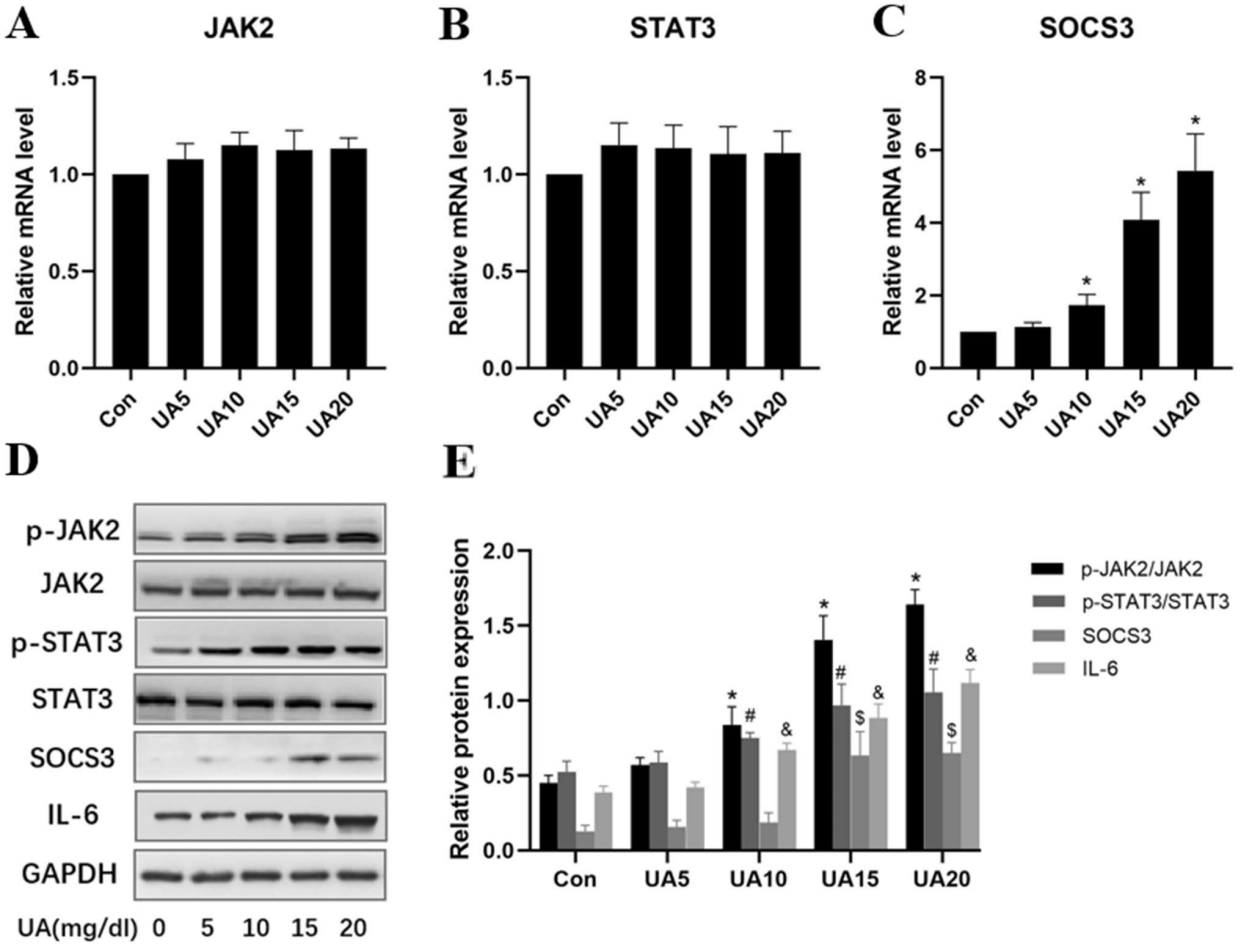
Uric acid triggers HUVEC dysfunction by inducing JAK2/STAT3 signaling. **A**–**C** Relative mRNA levels of JAK2/STAT3 signaling intermediates. **D**–**E** Relative protein amounts of JAK2/STAT3 signaling intermediates. p-JAK2/JAK2, p-STAT3/STAT3, SOCS3 and IL-6 were examined. *P < 0.05 vs. Con (p-JAK2/JAK2), ^#^*P* < 0.05 vs. Con (p-STAT3/STAT3), ^$^*P* < 0.05 vs. Con (SOCS3), ^&^*P* < 0.05 vs. Con (IL-6). Data are mean ± SE (n = 3)

**Fig. 5:**
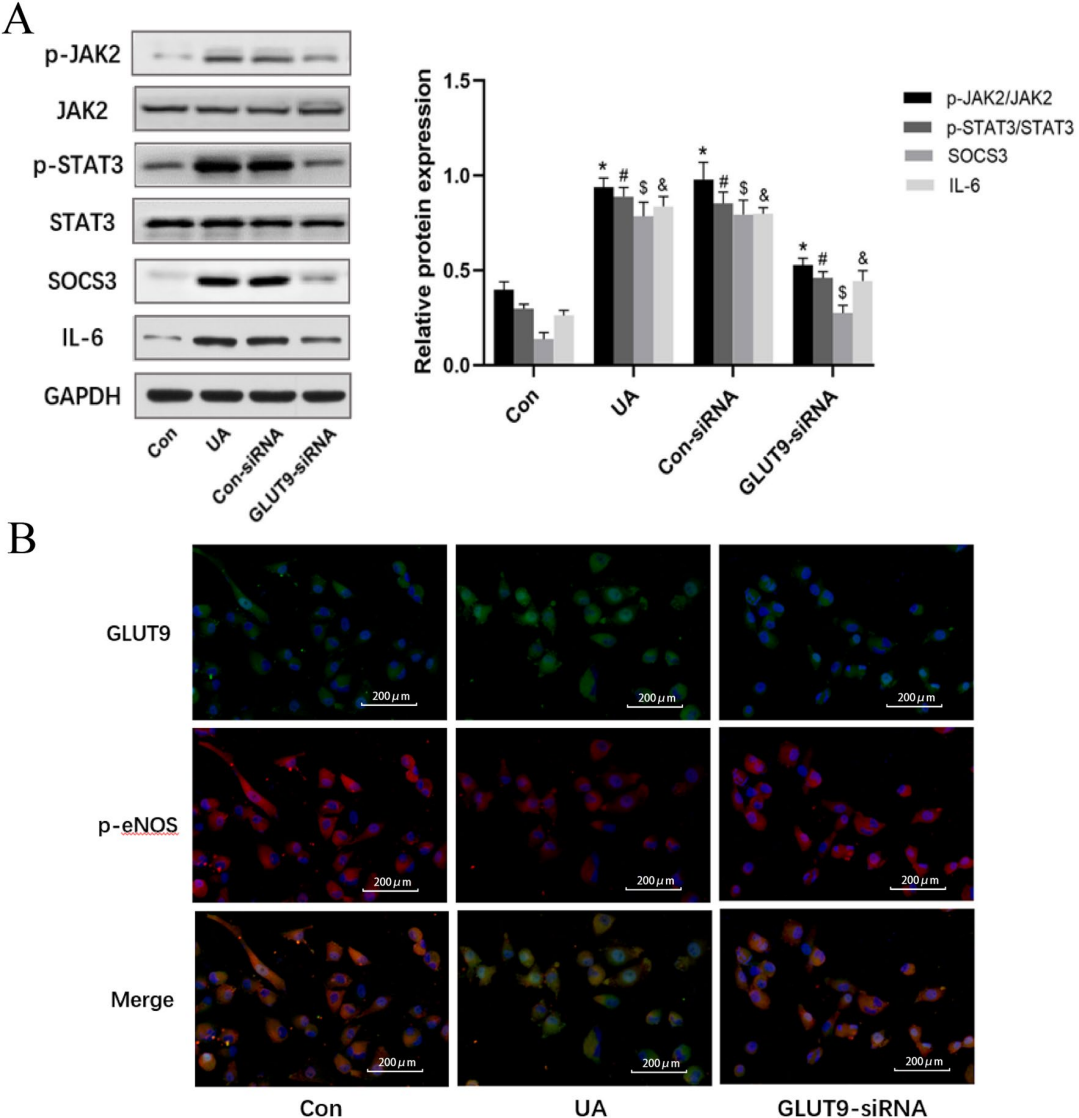
Effect of GLUT9 knockdown on the JAK2/STAT3 pathway and eNOS expression. **A** Relative protein amounts of JAK2/STAT3 signaling intermediates. *P < 0.05 vs. Con (p-JAK2/JAK2), ^#^*P* < 0.05 vs. Con (p-STAT3/STAT3), ^$^*P* < 0.05 vs. Con (SOCS3), ^&^*P* < 0.05 vs. Con (IL-6). Data are mean ± SE (n = 3). **B** Immunofluorescence for GLUT9 (green) and eNOS (red) in HUVECs (scale bars 200 μm). (Color figure online)

JAK2/STAT3 signaling activation is known to worsen cellular oxidative stress. Therefore, we aimed to determine whether UA increases intracellular ROS content by inducing JAK2/STAT3 signaling, subsequently leading to endothelial cell dysfunction and inflammation. The results showed that UA treatment had no effects on JAK2 and STAT3 mRNA amounts, but SOCS3 was significantly upregulated by UA at concentrations of 10, 15 and 20 mg/dl (Fig. 4A–C). Furthermore, phosphorylated JAK2 and STAT3 levels were dose-dependently elevated after UA treatment; IL-6 and SOCS3 protein amounts were increased by UA at concentrations of 15 and 20 mg/dl (Fig. 4D–E). However, knockdown of GLUT9 reduced phosphorylated JAK2 and STAT3 amounts (Fig. 5A) and enhanced eNOS phosphorylation in HUVECs (Fig. 5B).

## Discussion

Multiple mechanisms explaining UA’s role in endothelial dysfunction and cardiovascular diseases have been suggested. It was previously demonstrated UA increases calcium amounts in the mitochondria, which leads to greater production of mitochondrial superoxide, mitochondrial dysfunction, ROS generation [13, 14], lower eNOS expression, activation of the p38/ERK1/2 pathway, and endoplasmic reticulum stress in, and therefore damage to, endothelial cells [3, 15]. However, it remains unclear whether extracellular urate itself or intracellular accumulation of urate causes endothelial dysfunction. Here, HUVECs were treated with high UA concentrations to mimic hyperuricemia, and the roles played by the UA transporter GLUT9 in the deleterious effects of UA on HUVECs were evaluated, attempting to explore the molecular mechanisms involved.

Hyperuricemia was associated with lower plasma membrane expression of the UA efflux transporter BCRP, which leads to decreased UA efflux and intracellular UA accumulation [12]. In addition, elevated UA concentrations resulted in greater UA uptake via UA transporter-1 and greater production of cell adhesion factors [16]. As shown above, survival of HUVECs was decreased by UA only at a concentration of 20 mg/dl at the 24-h time point. Therefore, it seemed that UA did not have an acute effect on endothelial cell survival. We also determined that the uptake of UA increased dose- and time-dependently. Additionally, the UA transporter GLUT9 was upregulated by UA concentration-dependently, and GLUT9 knockdown reduced UA uptake. These data suggest that UA at a relatively high extracellular concentration upregulates GLUT9 to increase its uptake into endothelial cells.

NO release by endothelial cells plays a critical role in vascular homeostasis [17, 18]. In this study, UA had deleterious effects on endothelial cells: NO content, and eNOS activity and phosphorylation in HUVECs decreased with increasing UA concentrations. Additionally, MCP-1, ICAM-1 and VCAM-1 mRNA were increased, which implies increased endothelial cell inflammation. Previous reports demonstrated UA inhibits NO production by suppressing the phosphorylation of eNOS [7, 19]. However, UA is also considered an effective antioxidant that scavenges singlet oxygen and free radicals [20, 21]. As demonstrated above, 5 mg/dl UA had no significant effects on endothelial function and inflammatory response, but when the concentration was increased to 10 mg/dl, the inflammatory response increased significantly.

To explore the mechanism underpinning this effect, we examined GLUT9’s role. GLUT9’s effect on oxidative stress was determined by treating HUVECs with siRNA-GLUT9 and the antioxidant NAC, respectively. The results showed that siRNA and NAC treatment reduced intracellular ROS content, and GLUT9 expression was not inhibited when oxidative stress was relieved by NAC administration. Therefore, we can speculate that overexpression of GLUT9 would increase the accumulation of UA in cells and stimulate oxidative stress, which has never been addressed previously.

Inflammation is critical in initiating and sustaining vascular injury. Previous studies have shown that UA causes inflammation in endothelial cells, which manifests as upregulation and enhanced release of pro-inflammatory molecules, including IL-6, THF-α, IL-1β and MCP-1 [22]. However, the specific mechanism by which UA causes inflammation in vascular endothelial cells remains unclear. High UA amounts trigger inflammation and endothelial damage by activating NF-κB/ERK signaling [23]. In addition, UA crystals activate NLRP3 or interact with receptors on the cell membrane, including toll-like receptors 2/4, to mediate the inflammatory response [24–26]. Activation of JAK2/STAT3 signaling is known to cause oxidative stress [27, 28]. As shown above, JAK2/STAT3 was upregulated by UA in a concentration-dependent manner in HUVECs, as well as the pro-inflammatory cytokine IL-6. Knockdown of GLUT9 inhibited JAK2/STAT3 signaling, reduced the secretion of pro-inflammatory factors and upregulated eNOS in endothelial cells. These data indicate a very important role for GLUT9 in UA-dependent endothelial cell dysfunction.

## Conclusions

High UA levels induce endothelial cell dysfunction, which is characterized by decreased eNOS activity and NO production. These effects require GLUT9, which mediates the accumulation of UA in cells, which in turn activates the JAK2/STAT3 pathway, inducing inflammation and oxidative stress.

## Supplementary Information

Below is the link to the electronic supplementary material.

Supplementary file1 (PDF 102 kb)

Supplementary file2 (PDF 80 kb)

## Data Availability

The data are available from the corresponding author upon reasonable contact.
