# Supplementary material for: The effects of hyperuricemia on endothelial cells are mediated via GLUT9 and the JAK2/STAT3 pathway
Source: Mol Biol Rep. 2021 Oct 30;48(12):8023–32. doi: 10.1007/s11033-021-06840-w (PMC8604859; doi:10.1007/s11033-021-06840-w)
Supplement: Supplementary file 1 — Supplementary file1 (PDF 102 kb) [file 11033_2021_6840_MOESM1_ESM.pdf]

Table S1 Primer sequences used in RT-PCR

Gene	Forward primer (5′–3′)	Reverse primer (5′–3′)
eNOS	GCGAGTGAAGGCGACAATC	GGCCGGACATCTCCATCAG
MCP-1	CTCGCTCAGCCAGATGCAAT	CACTTGCTGCTGGTGATTCTTCT
ICAM-1	AGCTTCTCCTGC TCTGCAAC	GTCTGCTGGGAATTTTCTGG
VACM-1	TGTTCCAGCGAGGGTCTACCA	TCTCCAATCTGAGCAGCAATCC
GLUT9	GACTCCAGAGGGGCATGAAAA	AGCAGGACCAGTCCAATTTCT
JAK2	CCTGATGGATTACAAGGATGACG	CCTTCCACAAACTCTTCCACCAT
STAT3	GAGAAGGACATCAGCGGTAAG	CAGTGGAGACACCAGGATATTG
SOCS3	GCTCCAAGAGCGAGTACCAG	GTCACTGCGCTCCAGTAGAA
GAPDH	GGAAGCTTGTCATCAATGGAAATC	TGATGACCCTTTTGGCTCCC
