# Supplementary material for: The effects of hyperuricemia on endothelial cells are mediated via GLUT9 and the JAK2/STAT3 pathway
Source: Mol Biol Rep. 2021 Oct 30;48(12):8023–32. doi: 10.1007/s11033-021-06840-w (PMC8604859; doi:10.1007/s11033-021-06840-w)
Supplement: Supplementary file 2 — Supplementary file2 (PDF 80 kb) [file 11033_2021_6840_MOESM2_ESM.pdf]

Table S2 Sense and antisense sequences for siRNA

SiRNA	Sense (5′–3′)	Antisense (5′–3′)
GLUT9	GCAAUUUGGUUCUAUACCATT	UGGUAUAGAACCAAAUUGCTT
Control	UUCUCCGAACGUGUCACGUTT	ACGUGACACGUUCGGAGAATT
